# Determination of prophylactic and therapeutic effectiveness of probiotic strain *Escherichia coli* 39-SN

**DOI:** 10.25122/jml-2021-0118

**Published:** 2022-01

**Authors:** Birzhan Biyashev, Kadyr Biyashev, Madina Bulegenova, Zhumagul Kirkimbaeva, Arman Zhylkaidar

**Affiliations:** 1. Department of Microbiology, Virology, and Immunology, Kazakh National Agrarian Research University, Almaty, Republic of Kazakhstan

**Keywords:** newborn animals, gastrointestinal diseases, antibiotics, microbiocenosis, microbial preparations

## Abstract

At present, it is relevant to preserve and raise healthy, well-developed newborn animals adapted to new conditions, which form the basis for increasing the yield of animal husbandry. Gastrointestinal diseases cause the main losses of young animals. Acute gastrointestinal diseases of calves, lambs, piglets, and chickens are widespread in Kazakhstan. The study aims to develop a competitive treatment and prophylactic drug composition based on probiotic bacterial strains. Modern certified and standardized biochemical, microbiological, molecular biological studies were considered during the research. The morphological, cultural, and biochemical properties of the cultures were studied according to generally accepted schemes. Standard methods of finding averages and their mean errors were used for the mathematical processing of results. Antibiotics, sulfonamide, and nitrofuran drugs are the most common methods to combat diseases in young animals. However, the use of antibacterial agents often leads to the death of normal microflora, disrupting the microbiocenosis of the gastrointestinal tract, the appearance of microorganisms with resistance to drugs, and a decrease in product quality. In this regard, the direction of biotechnology involved in the development and creation of environmentally friendly microbial preparations with prophylactic effectiveness is very relevant. Data on the prophylactic and therapeutic efficacy of the probiotic strain of *Escherichia coli* 39-SN are presented.

## Introduction

Considering the disadvantages of probiotic drugs, the authors intended to develop a competitive treatment and prophylactic drug based on probiotic bacterial strains. When designing new probiotic preparations, it is necessary to consider quantities of various strains in the intestine, their physiological features, and their ability to survive in aggressive conditions (acidic stomach environment, bile acids) [1]. Furthermore, to increase the efficiency of preparations, it is important to select prebiotic correctly, an essential supplement for probiotic preparations, and a source of food for weakened representatives of intestinal microflora of the patient. Currently, the medical and veterinary services of many countries have a sufficient number of probiotic drugs of different species compositions aimed at correcting intestinal biocenosis and increasing the resistance of human and animal organisms. For example, only in Kazakhstan, 18 biological products of this class are registered and used in medical practice, and in the European Union (EU) countries, more than 20 probiotics. These drugs are different in composition, quality, pharmacological action, and indications for use [1–3]. However, monitoring the probiotic market shows that most developments are not in demand since, in some cases, probiotics do not correspond to the properties declared by the manufacturer. Sometimes the lack of effect leads to improper use and often discredits the probiotic they have worked with in a particular case and the entire direction.

It should be noted that most probiotics include strains isolated from the human intestine or taken from a collection of strains for food biotechnology. This applies to Bifidobacterium lactis, *Bifidobacterium bifidum, Bifidobacterium adolescentis, Bifidobacterium Longum, Lactobacillus acidophilus, Lactobacillus plantarum, Lactobacillus fermentum* etc. It is essential to consider that bifidobacteria, lactobacilli, and other microorganisms are not the same. The genus of bifidobacteria, for example, unites 24 species of microorganisms. The genus of lactobacilli is even larger. Some species live only in certain animals, others in many species of animals, others in animals and humans, and others in humans only. Accordingly, the ability to colonize the intestines in humans, animals, or birds is different. When selecting strains for probiotics, these and many other biological properties must be taken into account [4, 5]. Subsequently, the approach of developing probiotics should be based on the study of many parameters, including a comprehensive assessment of the properties of microorganisms – probionts. When selecting cultures for the preparation of probiotics, these must have a set of properties that allow them to compete with pathogenic and conditionally pathogenic microorganisms and meet certain requirements: be normal inhabitants of the gastrointestinal tract of healthy animals; non-pathogenic and non-toxic; transfer passage through the stomach (have a certain level of resistance to bile and hydrochloric acid); ability to adhere to the epithelium and engraftment in the digestive tract; antagonistic activity and be stable and able to remain viable for a long time when stored under industrial conditions [6–8].

## Material and Methods

The object of the study was the probiotic strain of *Escherichia coli* 39-SN, obtained through the genetic method. The strain *Escherichia coli* 39-SN was donated to the Collection of Microorganisms of the Republican State Enterprise Scientific Research Institute of Biological Safety of the Ministry of Education and Science of the Republic of Kazakhstan, Collector number M-46-15/D. The patent for the utility model No. 41 45 dated April 8, 2019, was obtained for the strain. In the experiment, virulent cultures of *E. coli* 15, S. *Typhimurium* 371, were used to infect experimental animals. The most important requirement for the manufacture and control of biological products is the production flow chart of the drug prepared from the *E. coli* 39-SN strain [9–12]. For the preparation of the *E. coli* strain 39-SN, the main digest of Hottinger was used, containing 10% of liver extract, 0.4% of peptone, then distilled water was added so that the content of amine nitrogen was at least 200–250 mg/% [13–16]. Modern certified and standardized biochemical, microbiological, molecular biological studies were considered during the research. The morphological, cultural, and biochemical properties of the cultures were studied according to generally accepted schemes [17]. The identification of selected cultures was conducted by the determinant of Bergie. During the experiments, laboratory animals, calves, lambs, piglets, and chickens were used for studying the pathogenicity of cultures isolated from dead, sick, and healthy birds. Standard methods of finding averages and their mean errors were used for the mathematical processing of results.

The effectiveness of probiotic strain *Escherichia coli* 39-SN on the total amount of protein and the quantitative and qualitative content of immunoglobulins in the blood serum of newborn calves was studied after a single oral administration of probiotic strain *Escherichia coli* 39-SN at doses of 2xl0^5^° colony-forming unit (CFU), 3xl0^10^ CFU and 4x10^10^ CFU for 20–30 min (before feeding colostrum) in a volume of 40 ml. The experiment involved 45 calves (10 calves for each dose and 15 in the control group, which were fed with saline). The total amount of protein and the quantitative and qualitative content of immunoglobulins were determined by the FT-2 automatic immuno-analyzer manufactured by the American Society for Microbiology (Italy). The objective of this research was to determine the prophylactic and therapeutic effectiveness of the strain *Escherichia coli* 39-SN in newborn animals and birds. The positive results from testing the *Escherichia coli* 39-SN strain in a laboratory model allowed us to test the strain on newborn calves, lambs, piglets, and chickens and then test it directly under production conditions. The prophylactic efficacy of the *E. coli* 39-SN strain was determined on newborn calves, lambs, piglets, and chickens by single oral feeding of animals in appropriate doses. After 24 hours, the titrated lethal dose was administered to the experimental and control animals in an orally virulent culture.

## Results

The research results are presented in Table 1. The study results indicate that the optimal effective prophylactic dose of the drug is 3x10^10^ CFU for calves, 10^10^ CFU for lambs, 10^10^ CFU for piglets, and 10^9^ CFU for chickens. At the same time, 100% safety of young animals is ensured upon infection with a virulent culture of *E. coli*.

**Table 1.** Determination of the optimal prophylactic dose of E. coli 39-SN.

**Animal**	**Number of animals**	**Dose introductions product Calves Calves Calves Calves Calves**	**The result of animal contagion by the virulent culture *E. coli***			
			**Infecting dosage, CFU**	**Died**	**Survived**	**Percentage survival rates**
**Calves**	10	10^10^	2x10^10^	2	8	80
**Calves**	10	2x10^10^	2x10^10^	1	9	90
**Calves**	10	3x10^10^	2x10^10^	-	10	100
**Calves**	10	4x10^10^	2x10^10^	-	10	100
**Calves (control)**	10	-	2x10^10^	10	-	-
**Lambs**	10	3x10^9^	10^10^	3	7	70
**Lambs**	10	5x10^9^	10^10^	1	9	90
**Lambs**	10	10^10^	10^10^	-	10	100
**Lambs**	10	2x10^10^	10^10^	-	10	100
**Lambs (control)**	10	-	10^10^	10	-	-
**Piglets**	10	3x10^9^	10^10^	2	8	80
**Piglets**	10	5x10^9^	10^10^	1	9	90
**Piglets**	10	10^10^	10^10^	-	10	100
**Piglets**	10	2x10^10^	10^10^	-	10	100
**Piglets (control)**	10	-	10^10^	10	-	-
**Chickens**	10	5x10^7^	10^9^	2	8	80
**Chickens**	10	5x10^8^	10^9^	1	9	90
**Chickens**	10	10^9^	10^9^	-	10	100
**Chickens**	10	2x10^9^	10^9^	-	10	100
**Chickens (control)**	10	-	10^9^	10	-	-

The therapeutic efficacy of *E. coli* 39-SN was determined on newborn calves, lambs, piglets, and chickens. Initially, the test animals were perorally injected with virulent cultures of Salmonella, Proteus, Klebsiella, Bacillus in infectious doses. On the 3^rd^ day (48 hours later), after introducing the virulent cultures, the experimental animals were given a single oral suspension of *E. coli* 39-SN. Control animals did not receive the drug. The results are shown in Table 2. As can be seen from the table, the *E. coli* 39-SN strain supports the recovery of calves, lambs, piglets, and chickens after infection with virulent cultures of Salmonella, Proteus, Klebsiella, Bacilli. The results indicate a high therapeutic efficacy and safety of the drug obtained from the strain *E. coli* 39-SN.

**Table 2.** Determination of the therapeutic efficacy of strain E. coli 39-SN.

**Type of animal**	**Number of animals**	**Culture Test (virulent)**		**Result of injection**				**Note**
		**Name**	**Infecting dosage, CFU**	**Infecting dosage, CFU**	**Died**	**Survived**	**Percentage survival rates**	
**Calves**	5	S. typhimurium	10^9^	3x10^10^	-	5	100	
**Calves**	5	S. typhimurium	10^9^	control	5	-	0	For 7–8 days
**Calves**	5	P. vulgaris	10^9^	3x10^10^	-	5	100	
**Calves**	5	P. vulgaris	10^9^	control	5	-	0	For 6–8 days
**Calves**	5	K. pneumonia	10^9^	3x10^10^	-	5	100	
**Calves**	5	K. pneumonia	10^9^	control	5	-	0	For 8–9 days
**Calves**	5	B.subtilis	10^9^	3x10^10^	-	5	100	
**Calves**	5	B.subtilis	10^9^	control	5	-	0	For 5–6 days
**Lambs**	5	S. typhimurium	10^8^	10^10^	-	5	100	
**Lambs**	5	S. typhimurium	10^8^	control	5	-	0	For 5–8 days
**Lambs**	5	P. vulgaris	10^8^	10^10^	-	5	100	
**Lambs**	5	P. vulgaris	10^8^	control	5	-	0	For 6–8 days
**Lambs**	5	K. pneumonia	10^8^	10^10^	-	5	100	
**Lambs**	5	K. pneumonia	10^8^	control	5	-	0	For 6–8 days
**Lambs**	5	B.subtilis	10^8^	10^10^	-	5	100	
**Lambs**	5	B.subtilis	10^8^	control	5	-	0	For 6–8 days
**Piglets**	5	S. typhimurium	10^8^	10^10^	-	5	100	
**Piglets**	5	S. typhimurium	10^8^	control	5	-	0	For 4-7 days
**Piglets**	5	P. vulgaris	10^8^	10^10^	-	5	100	
**Piglets**	5	P. vulgaris	10^8^	control	5	-	0	For 7–8 days
**Piglets**	5	K. pneumonia	10^8^	10^10^	-	5	100	
**Piglets**	5	K. pneumonia	10^8^	control	5	-	0	For 6–8 days
**Piglets**	5	B.subtilis	10^8^	10^10^	-	5	100	
**Piglets**	5	B.subtilis	10^8^	control	5	-	0	For 6–8 days
**Chickens**	10	S. typhimurium	10^5^	10^9^	-	5	100	
**Chickens**	10	S. typhimurium	10^5^	control	5	-	0	For 5–8 days
**Chickens**	10	P. vulgaris	10^5^	10^9^	-	10	100	
**Chickens**	10	P. vulgaris	10^5^	control	10	-	0	For 5–8 days
**Chickens**	10	K. pneumonia	10^5^	10^9^	-	10	100	
**Chickens**	10	K. pneumonia	10^5^	control	10	-	0	For 6–8 days
**Chickens**	10	B.subtilis	10^5^	10^9^	-	10	100	
**Chickens**	10	B.subtilis	10^5^	control	10	-	0	For 6–8 days

The positive results obtained from testing the preparation of *E. coli* 39-SN strain in the laboratory model and on the newborn calves, lambs, piglets, and chickens, formed the basis for further testing it directly in the production environment. The preparation of *E. coli* 39-SN strain was used for prophylactic purposes in the Bereke, Habit, and Almaty households, where gastrointestinal diseases were caused by pathogenic microbes of the intestinal group. The preparation of strain *E. coli* 39-SN was tested on 480 newborn calves, lambs, piglets, and chickens. The drug was used once before the first feeding no later than 30 minutes after birth using the following doses: calves – 3x10^10^ CFU, lambs – 10^10^ CFU, pigs – 10^10^ CFU, chickens – 10^9^ CFU. The results of the use of the drug are presented in Table 3.

**Table 3.** The results of the use of the preparation of E. coli 39-SN strain

**Household**	**Type of animal**	**Groups of animals**	**Number of animals**	**Died**		**Left**	
				**Animals**	**%**	**Animals**	**%**
Bereke	Calves	Experimental	30	-	-	30	100
		Control	10	3	30	7	70
	Lambs	Experimental	30	-	-	30	100
		Control	10	4	40	6	60
	Piglets	Experimental	30	-	-	30	100
		Control	10	2	20	8	80
	Chickens	Experimental	30	-	-	30	100
		Control	10	5	50	5	50
**Habit**	Calves	Experimental	30	1	3	29	97
		Control	10	4	40	6	60
	Lambs	Experimental	30	-	-	30	100
		Control	10	2	20	8	80
	Piglets	Experimental	30	2	6	28	94
		Control	10	3	30	7	70
	Chickens	Experimental	30	-	-	30	100
		Control	10	4	40	6	60
**Almaty**	Calves	Experimental	30	-	-	30	100
		Control	10	2	20	8	80
	Lambs	Experimental	30	-	-	30	100
		Control	10	2	20	8	80
	Piglets	Experimental	30	-	-	30	100
		Control	10	2	20	8	80
	Chickens	Experimental	30	-	-	30	100
		Control	10	3	30	7	10

## Discussion

One of the promising areas is the development of a probiotic preparation containing normal intestinal microflora – *Escherichia coli*, which in the process of life produces a complex of biologically active compounds that act on conditionally pathogenic microorganisms exerting antagonistic activity [9]. *Escherichia coli* began to be used as a basis for biological products at the beginning of the last century. Its effectiveness was explained by replacing toxic bacteria with normalized intestinal microflora. *Escherichia coli* based preparations were mainly used to treat and prevent intestinal dysbiosis [10–12]. Probiotic microorganisms must be able to multiply in the gastrointestinal tract actively. They can produce biologically active metabolites resistant to gastric juice and bile. Probiotics should not have contraindications for use and should not cause adverse reactions in the body after administration [13]. The primary task of the Kazakh production of probiotics intended to maintain and restore the symbiotic microbiocenoses of humans and animals is the production of competitive drugs that are not inferior in their consumer properties to similar imported drugs [14, 15].

Probiotics have multiple effects on the body of an animal. As normal inhabitants of the gastrointestinal pathway, probiotic drugs normalize the microbial landscape, affecting metabolic processes. In addition, the drugs have an immunostimulating, growth-stimulating, preventive effect by inhibiting the growth of pathogenic microorganisms as a result of the production of antimicrobial and biologically active substances, stimulating the growth of normal microflora, altering microbial metabolism, synthesizing vitamins and other growth-stimulating substances, normalize pH and neutralize toxins [18–21].

## Conclusions

The results of industrial testing showed that the *E. coli* 39-SN strain significantly reduces the incidence of disease in calves, lambs, piglets, and chickens, improves their general condition, and provides 94–100% safety of young animals with 30–40% death of control animals. The tests showed the possibility and high efficiency of its use in veterinary medicine. It inhibits pathogenic enterobacteria, maintains optimal microbial balance in the digestive tract, increases non-specific resistance of animals, their safety, and weight gain, and has a preventive and therapeutic effect in diseases accompanied by diarrhea and prevention dysbacteriosis. Summing up, the authors believe that the positive results obtained from testing the preparation of the *E. coli* 39-SN strain both on the laboratory model and on newborn calves, lambs, piglets, and chickens are the basis for further widespread use in farms of the Republic of Kazakhstan as a prophylactic drug against intestinal infections among young farm animals and birds.

## Acknowledgments

### Conflict of interest

The authors declare no conflict of interest.

### Ethical approval

All procedures performed in the study followed the ethical standards of the institutional and national research committee and the 1964 Helsinki declaration and its later amendments or comparable ethical standards. This study was approved by the Kazakh National Agrarian Research University, No. 1457.

### Authorship

BB contributed to project administration, conceptualization, methodology, investigation, writing, review, editing, visualization, supervision. KB and MB contributed to the conceptualization, methodology, investigation, data curation, writing, review, editing, visualization. ZHK, AZH contributed to formal analysis, investigation, resources, data curation, writing, review, editing, supervision.
